# Environmental DNA-based xenomonitoring for determining *Schistosoma* presence in tropical freshwaters

**DOI:** 10.1186/s13071-020-3941-6

**Published:** 2020-02-12

**Authors:** Hind Alzaylaee, Rupert A. Collins, Asilatu Shechonge, Benjamin P. Ngatunga, Eric R. Morgan, Martin J. Genner

**Affiliations:** 1 School of Biological Sciences, University of Bristol, Life Sciences Building, 24 Tyndall Avenue, Bristol, BS8 1TQ UK; 2 Biology Department, Faculty of Sciences, Prince Nourah Bin Abdulrahman University, Riyadh, Saudi Arabia; 3 Tanzania Fisheries Research Institute (TAFIRI), PO Box 9750, Dar es Salaam, Tanzania; 4 School of Biological Sciences, Queen’s University Belfast, 19 Chlorine Gardens, Belfast, BT9 5DL UK

**Keywords:** Schistosomatidae, Freshwater eDNA, African freshwater body, Detection and quantification level

## Abstract

**Background:**

Schistosomiasis is a neglected tropical disease that infects over 200 million people worldwide. Control measures can benefit from improved surveillance methods in freshwaters, with environmental DNA (eDNA) surveys having the potential to offer effective and rapid detection of schistosomes. However, sampling eDNA directly from natural water bodies can lead to inaccurate estimation of infection risk if schistosome eDNA is rare in the environment. Here we report a xenomonitoring method that allows schistosome infections of host snail species to be determined from eDNA in water used to house those snails.

**Methods:**

Host snail species were collected and placed in containers of water and allowed to shed cercariae, and then water samples were filtered and tested using qPCR assays specific to the African species *Schistosoma mansoni* and *Schistosoma haematobium*. We evaluated this “eDNA-based xenomonitoring” approach by experimentally comparing the results to those obtained from direct qPCR screening of tissue sourced from the snails in the experiment.

**Results:**

We found that our method accurately diagnosed the presence of *S. mansoni-*infected snails in all tests, and *S. haematobium-*infected snails in 92% of tests. Moreover, we found that the abundance of *Schistosoma* eDNA in experiments was directly dependent on the number and biomass of infected snails.

**Conclusions:**

These results provide a strong indication that this surveillance method combining the utility of eDNA-based monitoring with the reliability of traditional xenomonitoring approaches could be used to accurately assay the presence of *Schistosoma* species in natural habitats. This approach may be well-suited for epidemiological studies and monitoring in endemic areas, where it can assist schistosomiasis control by indicating infection risk from freshwaters and guiding necessary interventions to eliminate the disease.
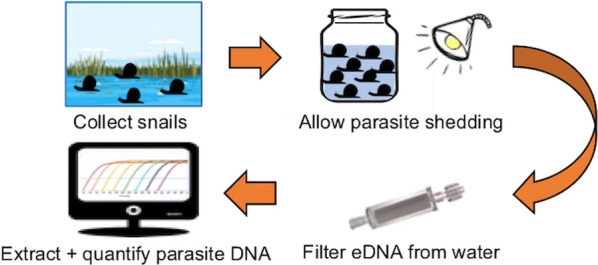

## Background

Schistosomiasis, also known as snail fever or bilharzia, affects an estimated 207 million people in over 67 countries worldwide, and there are over 779 million further people at risk of infection [1]. The disease is considered a major cause of disability impeding socioeconomic development in regions of the world where it is endemic [2]. It is listed as a ‘neglected tropical disease’ and has been recognised by the World Health Assembly as a disease that should be targeted by control programmes and elimination campaigns where appropriate [3]. The disease is caused by parasitic trematodes that as adults are present in the blood vessels surrounding the urogenital or gastrointestinal tracts of human hosts. Eggs are then released into freshwaters *via* urine and faeces, miracidia hatch from eggs and infect snail hosts. Infected snails later release cercariae into the water, and the disease is acquired by humans when they encounter the cercariae [2]. While the disease can be treated in humans using anthelmintic medication, a key factor in elimination of the disease will be the prevention of reinfection after treatment [3–6]. This could be achieved by reducing exposure of the human population to free-swimming schistosome cercariae, either by treating or manipulating freshwater habitats to eliminate snail hosts [7], or by alerting local human populations to the infection risk associated with use of freshwater environments. Both strategies will require an appropriate surveillance framework for the presence of schistosomes in freshwaters [8]. Moreover, expansion of areas suitable for transmission under climate change requires pro-active monitoring of new at-risk areas [9].

Conventionally, environmental monitoring for *Schistosoma* spp. has primarily focused on snail-based surveys in which snails are collected and exposed to light to induce cercarial shedding. Microscopical examination of schistosome cercariae is then used to determine the infection status of snails [10–12], and the method requires considerable time, effort and expertise in the taxonomic identification of schistosome cercariae using microscopy. Alternatively, it is possible to test infection status of individual snails using molecular xenomonitoring tests for the presence of *Schistosoma* DNA in snail tissue using conventional end-point PCR [13–16] or quantitative PCR [17, 18]. While these methods requiring testing of individual snails have been very effective, they are limited by the need to test large numbers, as often only a small proportion of a total snail population are infected [19, 20]. Thus, without extensive testing using conventional methods it is possible for a schistosomiasis-endemic area with a low parasite burden to appear free of the infection risk, but transmission may continue with the potential to expand in future [20].

Movement towards tests that can rapidly and reliably assess infection risk from trematode parasites in natural water bodies has been achieved through “cercariometry” (the collection and molecular testing of free-swimming cercariae) [21, 22], or testing of environmental DNA (eDNA) sampled directly from freshwaters [23, 24]. Methods sampling “environmental DNA” are varied, but from a parasitological perspective, the term eDNA has been defined as “DNA extracted from environmental or organismal matrices, in other words from the environment or host organism” [25]. Because environmental samples are inherently heterogeneous, it is therefore appropriate from this parasitological standpoint to define any DNA from microscopic organisms present within environmental samples to be eDNA, irrespective of whether the DNA sampled originated from whole microscopic organisms, cellular debris, chemically bound DNA, or DNA in solution.

For schistosome surveillance, eDNA methods that rely on screening water samples collected directly from natural environment are promising, given the relative ease of field sampling, and the absence of any firm requirements to directly sample living organisms. However, the use of eDNA from such samples still needs evaluation, particularly in cases where infected snails are rare and *Schistosoma* DNA is consequently below the limits of detection. Assays could yield false negatives if the water is turbid and adequate water volumes cannot be filtered, if turbid water contains PCR inhibitors, or if water movement transports eDNA away from a sampling site. Moreover, where *Schistosoma* eDNA is detected in areas where no snails were found during manual searches [24], it can be uncertain if infected snails are present but went undetected, or if parasite material has been transported to the sampling site from elsewhere. It is also possible that the local environment contains schistosome material released as miracidia from infected mammalian hosts, but the infectious cercarial stage is absent [24].

**Fig. 1:**
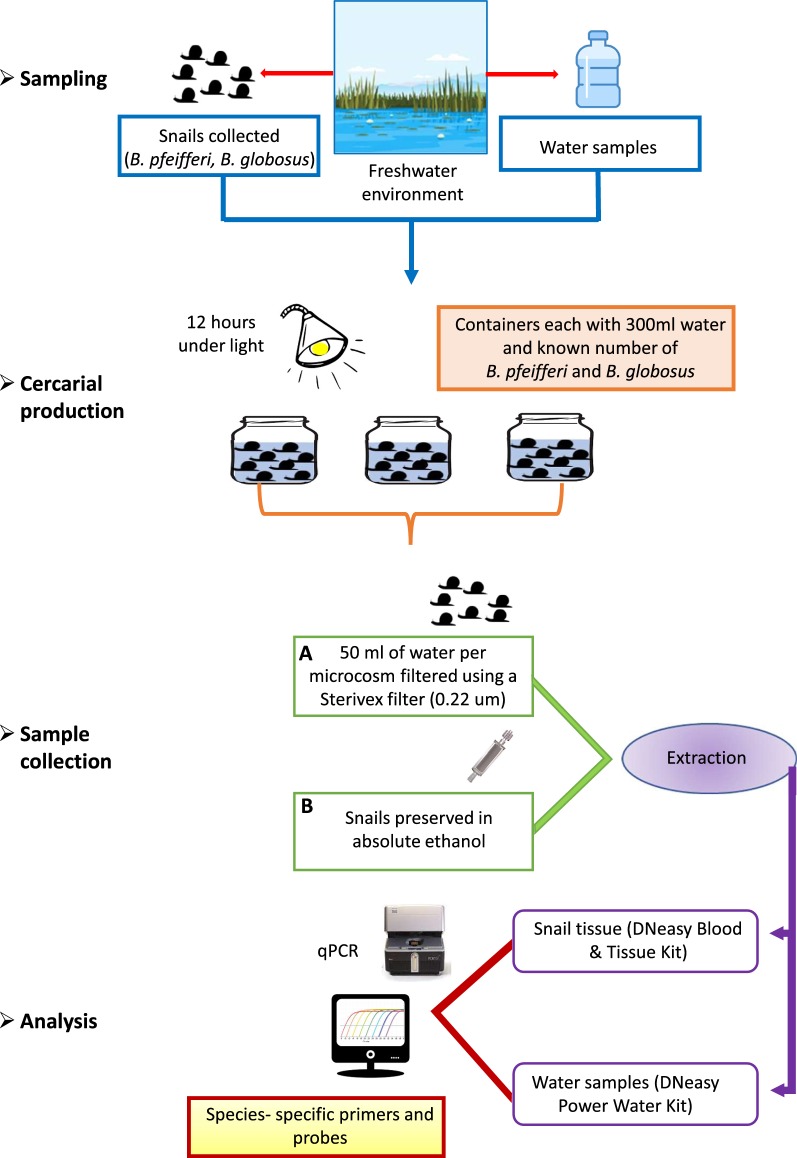
Overview of steps in the eDNA-based xenomonitoring assay, from sampling to analysis. Validation of the assay is conducted using qPCR analysis of tissue from preserved snails

Given the potential limitations of testing for the presence of *Schistosoma* species using eDNA collected directly from the sampling site, particularly the risk of false negatives in cases of low schistosome density, it would be beneficial to design a protocol that enables the schistosome eDNA originating from cercariae shed by snail hosts to be concentrated prior to molecular testing. In this study, we report an approach where snails are collected and housed in experimental containers to allow them to shed cercariae, before eDNA in the water - from whole cercariae, cellular debris, or DNA chemically bound or in solution - is collected and its abundance measured using quantitative PCR (Fig. 1).

## Methods

### Site description

Tanzania is a country with high schistosomiasis endemism, where primary intermediate hosts include the freshwater snails *Biomphalaria pfeifferi* for *S. mansoni* and *Bulinus globosus* for *S. haematobium*. On 16th September 2018, potential schistosome host snails were collected from two proximate locations in the Mpemba River in the Lake Rukwa catchment. At the time of the sampling, Site 1 (9.242°S, 32.841°E) was slowly flowing, and shallow (1.0 m maximum depth), with a temperature of 23.3 °C, pH 8.15, and conductivity 300 μS. Site 2 (9.265°S, 32.841°E) was not flowing, shallow (0.5 m maximum depth) with a temperature of 27.5 °C, pH 8.67, and conductivity 430 μS. At both sites the schistosome host snails *B. pfeifferi and B. globosus* were present, and snails were collected by scooping along 50 m of river. These sites were chosen as they are within an endemic area of *S. haematobium* and *S. mansoni*, and our pilot work had indicated that both species were present in the river. Moreover, both sites are near towns, and at the time of sampling there was clear evidence that the river was being used by the local population regularly for bathing, fishing, washing clothes, washing vehicles and collection of water for household activities.

### Experimental design and sample collection

All field-collected snails were taken back to the laboratory and identified to the species level based on shell morphology [26]. At the start of the experiment the infection status of individual snails was not known. The experiment included five different treatments (A–E) that differed in the number of snails kept in each container and used river water collected from Site 1 (Table 1). The design aimed to achieve the objective of a range of infected snail numbers and infected snail biomass across containers in the experiment as a whole. Specifically, Treatment A included a median of 20 *B. pfeifferi* and 3 *B. globosus*, Treatment B included a median of 20 *B. pfeifferi* and 6 *B. globosus*, Treatment C included a median of 10 *B. pfeifferi* and 3 *B. globosus*, Treatment D included a median of 10 *B. pfeifferi* and 6 *B. globosus*, and Treatment E was a negative control of river water that contained no snails (Table 1). In addition, Treatment F was a negative control treatment of tap water that contained no snails.

**Table 1:** Summary of the number of replicates in each treatment, and the numbers of snails in used in each of the treatments A-D (that were all housed in water from Site 1)

Treatment	No. of replicates	No. of *B. pfeifferi* Median (range)	Location source of *B. pfeifferi*	No. of *B. globosus* Median (range)	Location source of *B. globosus*
A	6	20 (19–21)	Site 1	3 (2–3)	Site 1
B	6	20 (19–21)	Site 1	6 (3–6)	Site 1
C	6	10 (10–11)	Site 1	3 (3–3)	Site 2
D	6	10 (10–11)	Site 1	6 (5–6)	Site 2
E^a^	6	–	–	–	–
F^b^	2	–	–	–	–

^a^Treatment E was a negative control (using water from Site 1)

^b^Treatment F was a negative control (using tap water)

*Notes*: There is some slight variation in the numbers of snails in each replicate, as on inspection after the experiment some snails used were found to be empty shells containing only sediment, while some small snails had tucked into shells of larger snails

Each of the six replicates within each of the river water treatments (A–E) included the use of one clear plastic sealable container (bottle with lid) filled with 300 ml of water collected from Site 1 approximately 12 h prior to the start of the experiment. Although it is possible that trace eDNA was present in this river water, it was necessary to use water from the natural environment to reduce the likelihood that snails and schistosomes would be negatively affected by substantive changes to the physio-chemical parameters of the water. Treatment F comprised two replicates each with one clear plastic sealable container (bottle with lid) filled with 300 ml of tap water.

After snails were introduced to containers, they were placed under artificial light, to induce cercarial shedding. After a period of 12 h, one 50 ml water sample was taken from each container using a sterile 50 ml syringe and filtered using a Sterivex filter with a pore size 0.22 µm and a polyethersulfone membrane (Merck, Darmstadt, Germany). To preserve the samples, absolute ethanol was passed through the filter with a sterile syringe. Post-filtering, Whirl-Pak bags (118 ml capacity; Nasco, Fort Atkinson, USA) were used to keep each filter separate and reduce potential contamination. After filtering the water, snails were preserved in absolute ethanol; each group in one bag. All field-collected samples (eDNA and snails) were kept as cool as possible in the field, and transported to the UK where they were stored at − 20 °C until DNA extraction. This eDNA-based method will potentially collect DNA from whole schistosomes, chemically bound or free DNA in solution in the environment, or DNA within cellular debris in the environment. In practical terms, however, the result is a measure of parasite DNA present in the immediate environment of the snail host, following augmentation by stimulation of cercarial release.

### DNA extraction from Sterivex filters

eDNA from filter samples was extracted individually using the DNeasy Power Water Kit (Qiagen, Venlo, Netherlands) following the manufacturer’s protocol. Before the extraction process, the laboratory bench was cleaned with 10% bleach, then with 70% ethanol, and finally UV light was used to eliminate residual DNA. All tools used for cutting and handling the filter, including blades, tweezers and scissors were wiped with 10% bleach then washed with 70% ethanol to avoid sample cross-contamination. Used gloves were exchanged for new at each step of the extraction process. Extraction of eDNA and DNA from tissue samples was carried out in different laboratories.

### DNA extraction from molluscs

Prior to extractions, snails were separated, and individual snails were washed with distilled water. The length (mm) and wet weight (g) of each snail was then measured. A small sample of tissue (no more than 20 mg), and DNA was extracted using the DNeasy Blood & Tissue Kits (Qiagen) according to the manufacturer’s protocol.

### eDNA assay of samples

DNA quantification of eDNA samples used a qPCR approach based on the mitochondrial *16S* rRNA gene of *S. mansoni* and *S. haematobium.* Reactions were performed in a 5 µl final volume, containing 1 µl DNA template, 2.5 µl Master Mix (PrimeTime Gene Expression Master Mix; Integrated DNA Technologies, Coralville, IA), 1.25 µl molecular grade water (VWR International, Leicestershire, UK) and 0.25 µl of the primer/probe premix. The primer/probe premix was prepared with 4 µl of each primer (100 µM, Integrated DNA Technologies), 2 µl of probe (100 µM, Integrated DNA Technologies), and 40 µl of molecular grade water. The species-specific forward and reverse primers and probes are shown in Table 2. The qPCR conditions were as follows: 3 min at 95 °C for initial denaturation, followed by 45 cycles of 95 °C for 0.05 s and 60 °C for 30 s. Each sample was run in triplicate (technical replicates) and each plate of samples quantified with a 7-fold serial dilution of a control positive DNA sample (ranging from 1,000,000 copies/μl to 1 copy/μl), and a no-template negative control. The reactions were run on a Eco48 thermal cycler machine (PCRMax, Staffordshire, UK) in 48-well plates with ROX normalisation. DNA detection was expressed by quantification cycle threshold (Cq) values. We report the theoretical limits of detections as estimated as the number of copies at which there was a 95% probability of amplification in any one PCR of a sample (termed LOD_I_) and the 95% probability of amplification in any one of three PCRs of a sample (termed LOD_III_), using the standard dilution series, and fitting logistic models [27] using CurveExpert Basic 2.1.0 (Hyams Development). The theoretical limit of quantification (LOQ) was estimated as the minimum dilution in which 90% of standards amplified reliably [28].

**Table 2:** Details of species-specific assays for *S. mansoni* and *S. haematobium*

Species	Primer or probe	Sequence (5′-3′)	Product length (bp)
*S. mansoni*	Forward primer	CTGCTCAGTGAAGAAGTTTGTTT	104
	Probe	AGCCGCGATTATTTATCGTGCTAAGGT	
	Reverse primer	CCTCATTGAACCATTCACAAGTC	
*S. haematobium*	Forward primer	AATGAACATGAATGGCCGCA	143
	Probe	TGGAGACTTGTGAATGGTCGAACG	
	Reverse primer	ATGGGTTCCTCACCACTTAAACT	

*Notes*: Probes were designed with a dual labelled 5(6)-carboxy-fluorescein (FAM) fluorescent tag at the 5′ end and with a black hole quencher 1 (BHQ1) at the 3′**-**end

Tests for the presence of *S. mansoni* and *S. haematobium* within the tissue samples of *B. pfeifferi* and *B. globosus* were also conducted using the same qPCR approach as described above. However, in these tests only the presence or absence of amplification was recorded for samples, alongside positive and no-template negative controls, and DNA was not formally quantified using a dilution series.

### Data analysis

Cq values, DNA concentrations and the qPCR quantification parameters *r*^2^ and percent efficiency were calculated using EcoStudy version 5.2.16 (PCRMax) using the default settings. Data for each assay (*S*. *haematobium* and *Bulinus*; *S. mansoni* and *Biomphalaria*) were analysed separately using linear models in R 3.6.0 (R Core Team, 2019). Each model included the eDNA quantity as the response variable (measured as the mean number of eDNA copies across the technical replicates). The predictor variables were either the number of infected host snail individuals or the total infected host snail biomass in experimental replicates, as determined from the qPCR assays of snail tissue.

## Results

The qPCR efficiency of *S. mansoni* eDNA assay was 103% across the four dilution series assays (range 91.55–110.86%) with a mean *r*^2^ value of 0.99 (range 0.97–0.99). For *S. mansoni* the LOD_I_ was 32.36 copies/μl, the LOD_III_ was 1.49 copies/μl, and the LOQ was 100 copies/µl. No amplifications were observed in the no-template qPCR controls, the negative control samples of water collected from the natural water body, or from the local tap water controls. The tests revealed the presence of *S. mansoni* eDNA in water from all 24 containers with *Biomphalaria* host snails, with all 72 qPCR replicates showing positive amplifications (Additional file 1: Table S1). In total *S. mansoni* was present in 145 of 364 *Biomphalaria* individuals from the experiment that had their tissue tested. There was complete congruence between the presence of *S. mansoni* in the eDNA assay and the presence of *S. mansoni* in the tissue of the *Biomphalaria* host snails (Table 3).

**Table 3:** Results of *S. mansoni* and *S. haematobium* DNA experimental analyses

Treatment	Replicate code	*S. mansoni*					*S. haematobium*				
		eDNA amplification success	eDNA Cq (mean)	eDNA copies/µl (mean)	*n*/*N*	Infected snail biomass (g)	eDNA amplification success	eDNA Cq (mean)	eDNA copies/µl (mean)	*n*/*N*	Infected snail biomass (g)
A	1	3/3	32.47	211.48	8/21	1.49	3/3	32.88	20.14	3/3	1.38
	2	3/3	35.13	35.62	10/21	1.71	3/3	34.53	6.35	3/3	1.44
	3	3/3	33.92	72.73	6/19	0.83	2/3	36.11	2.64	2/3	1.03
	4	3/3	37.11	6.71	5/20	0.59	3/3	32.36	29.30	2/3	0.76
	5	3/3	38.59	2.57	12/21	1.91	0/3	–	0	1/2	0.38
	6	3/3	38.48	2.45	3/20	0.37	2/3	37.28	1.03	0/3	0
B	7	3/3	36.55	10.20	9/20	0.56	3/3	28.91	325.97	4/6	2.07
	8	3/3	34.47	52.78	6/20	1.07	2/3	36.28	1.95	1/3	0.33
	9	3/3	35.03	33.27	11/20	1.72	3/3	33.10	19.53	6/6	2.48
	10	3/3	33.85	77.28	7/19	0.84	3/3	29.56	222.73	4/6	1.68
	11	3/3	31.47	435.27	11/21	1.2	3/3	30.07	160.39	3/6	1.17
	12	3/3	33.59	94.77	6/20	0.76	3/3	33.10	19.50	1/6	0.49
C	13	3/3	36.14	18.16	4/10	0.64	3/3	34.86	8.01	2/3	0.53
	14	3/3	34.81	40.36	3/10	0.39	0/3	–	0	2/3	0.64
	15	3/3	39.68	1.09	4/10	0.30	3/3	37.09	1.48	3/3	0.78
	16	3/3	34.05	67.49	2/11	0.14	3/3	35.05	4.95	2/3	0.59
	17	3/3	36.21	22.03	3/10	0.37	3/3	24.17	8445.25	3/3	0.48
	18	3/3	36.03	20.46	5/10	0.54	3/3	28.77	342.91	3/3	1.20
D	19	3/3	35.03	43.35	4/10	0.66	3/3	34.29	7.61	5/5	1.23
	20	3/3	35.89	26.35	4/10	1.13	3/3	29.65	180.51	6/6	1.44
	21	3/3	37.26	12.39	7/10	0.97	3/3	31.49	49.80	5/5	1.07
	22	3/3	35.63	27.94	6/11	1.27	3/3	27.90	611.29	6/6	1.79
	23	3/3	37.95	5.25	4/10	0.58	3/3	35.36	3.49	6/6	1.44
	24	3/3	36.04	21.65	5/10	0.44	3/3	31.36	53.09	6/6	1.34
E	25	0/3	–	–	–	–	0/3	–	–	–	–
	26	0/3	–	–	–	–	0/3	–	–	–	–
	27	0/3	–	–	–	–	0/3	–	–	–	–
	28	0/3	–	–	–	–	0/3	–	–	–	–
	29	0/3	–	–	–	–	0/3	–	–	–	–
	30	0/3	–	–	–	–	0/3	–	–	–	–
F	31	0/3	–	–	–	–	0/3	–	–	–	–
	32	0/3	–	–	–	–	0/3	–	–	–	–

*Note*: The table includes the results from eDNA monitoring and the results of the qPCR tests of host snail infection

*Key*: –, no amplification was observed

*Abbreviations*: n, number of infected snails; N, number of all host snails in the assay

The qPCR efficiency of *S. haematobium* assay was 101% across the four dilution series assays (range 91.7–102.33%) with a mean *r*^2^ value of 0.99 (range 0.98–0.99). For *S. haematobium* the LOD_I_ was 1.33 copies/μl, LOD_III_ was ≤ 1 copy/μl while the LOQ was 100 copies/μl. Again, no amplifications were observed in the no-template qPCR controls, the negative control samples of water collected from the natural water body, or from local tap water controls. The results also revealed the presence of *S. haematobium* eDNA in water from 22 of the 24 experimental containers with *Bulinus* host snails, with 61 of 72 eDNA qPCR replicates showing positive amplifications (Additional file 1: Table S1). In total, *S. haematobium* was present in 79 out of 102 *Bulinus* individuals that had their tissue tested. There was a strong congruence between the presence of *S. haematobium* in the eDNA assay and the presence of *S. haematobium* in the tissue of the *Bulinus* host snails, with only three exceptions. Two experimental containers negative for *S. haematobium* eDNA contained *B. globosus* snails positive for *S. haematobium* in tissue assays, and one experimental container that was positive for *S. haematobium* eDNA contained *B. globosus* found negative for *S. haematobium* in the tissue assay (Table 3).

**Fig. 2:**
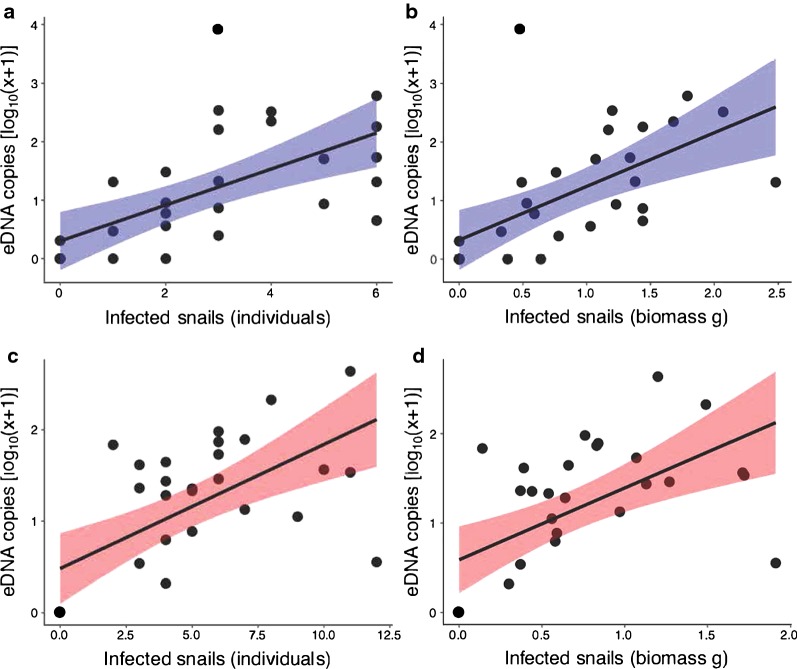
Associations between eDNA copies and infected snails in experimental containers. **a***Schistosoma haematobium* copies and number of infected host snail individuals. **b***S. haematobium* copies and biomass of infected host snail individuals. **c***S. mansoni* copies and number of infected host snail individuals. **d***S. mansoni* copies and biomass of infected host snail individuals. Lines illustrate linear models of associations between the variables, with 95% confidence intervals

There were strong associations between the number of infected snails and eDNA copy number; as the number of infected snails in an experiment increased, the number of eDNA copies increased (Fig. 2, Table 4). We also found strong associations between the infected biomass of host snails and eDNA abundance in the experimental containers, measured using eDNA copies. As the infected snail biomass increased, eDNA copy number increased (Table 4).

**Table 4:** Summary linear models, predicting the number of eDNA copies/µl (log_10_ transformed)

Assay	Predictor variable	Estimate (SE)	*F*_(1, 28)_	*r*^2^	*P-*value
*S. haematobium*	Infected host individuals	0.308 (0.073)	4.220	0.389	< 0.001
	Infected host biomass	0.916 (0.230)	3.980	0.361	< 0.001
*S. mansoni*	Infected host individuals	0.136 (0.032)	4.257	0.393	< 0.001
	Infected host biomass	0.804 (0.209)	3.854	0.347	< 0.001

*Abbreviations*: SE, standard error

## Discussion

Our results confirmed a strong association between the detection of DNA in the experimental chamber and the presence of infected snails for both *S. haematobium* and *S. mansoni* assays. One experimental replicate detected *S. haematobium* eDNA, but no *S. haematobium* in the tissue. This may be explained by schistosome life-cycle stages being absent from the specific sub-sample of tissue used, or alternatively it may be linked to PCR inhibition given potential for polysaccharides in mollusc tissue to act as PCR inhibitors [29]. Additionally, two experimental replicates failed to detect *S. haematobium* eDNA, but did detect *S. haematobium* in the tissue. This may be explained by the parasite DNA concentration in water sample being below the estimated level of detection with three qPCR technical replicates, or because of a failed DNA extraction, or perhaps cercariae were not shed into the surrounding water. We did not measure the number of snails actively shedding cercariae, but they can be absent if infection is prepatent [30–32], or if cercariae are affected by environmental factors such as interactions with other organisms. For example, rotifers can limit cercarial motility and infectivity, which may influence detectability [33, 34]. Nevertheless, despite these modest inconsistencies in results of the two testing methods, we clearly demonstrated that both numbers and biomass of infected host snails were significantly positively related to eDNA abundance, thus demonstrating that it is possible to use eDNA abundance to predict the number of infected host snails within containers used in the assay.

The results presented show for the first time that standardised eDNA-based xenomonitoring assays using consistent conditions can provide quantitative information on infection prevalence within snail populations sampled in the field, and therefore if coupled with snail abundance information it may be possible to quantify transmission risk. In principle, this method could be more consistent and reliable than direct eDNA sampling of water, which may depend on extrinsic factors, such as water flow, temperature and light regime in the days prior to sampling. Moreover, it would also be less labour-intensive than testing individual snails in order to quantify the prevalence of infection by conventional microscope-based identification of emerging cercariae [10–12]. Our method would also overcome potential taxonomic complications due to the sympatric coexistence of human and non-human schistosome species with morphologically similar cercariae [35, 36]. Additionally, it may be preferable to the PCR-based approaches on snail tissue (either end-point PCR [15] or qPCR as in this study), and loop-mediated isothermal amplification (LAMP) on snail tissue [37], given the time-consuming nature of DNA extraction from multiple individual snails, and the potential presence of polysaccharide PCR inhibitors in mollusc tissue [29]. More specifically, this eDNA-based xenomonitoring method that requires only one eDNA extraction from the water in each experimental chamber could replace the need to extract DNA individually from potentially hundreds of snails, thus providing significant savings in cost and time. In comparison to the subsampling and homogenising of large numbers of snails prior to DNA extraction, water sampling with enclosed filters can also represent an advantage in the laboratory in terms of speed, cost, and reduced likelihood of cross-contamination.

A practical challenge faced during the collection of eDNA samples is rapid blocking of filters from the turbid water that is typical of *Schistosoma* transmission sites. Typically, it is necessary to use fine small pore sizes (0.22 μm) [24, 38] and this can mean that it is only practical to sample 500 ml of water, or often much less, per filter. However, we note that large pore size units (350 μm) for pre-filtering water samples have been used successfully [24]. Nevertheless, the xenomonitoring method we used overcame these difficulties as it allowed sediment to settle prior to sampling. It also required only smaller (50 ml) volumes of water, as schistosome eDNA would have been more concentrated in our experimental containers than it would be in the natural environment. In principle, it would also be possible to precipitate eDNA directly from water samples, therefore foregoing the need to use filters [39, 40].

One of the key advantages of eDNA sampling directly from the environment is that it does not require collection or analysis of snails. By contrast, eDNA-based xenomonitoring, like conventional tests of the presence of cercariae, requires both sampling of snails and housing them in controlled conditions for the duration of an experiment. In practice, where snail infection is low, it may be necessary to collect and test several hundred snails to reach detectable levels of infection, but this could be readily achieved within experimental containers if sufficient numbers can be collected from the natural environment. Further research will be required to determine the relative power of different analytical approaches for detecting and quantifying schistosome prevalence across natural densities of snails and across differing intensities of snail infection. Specifically, it would be useful to compare the detection probabilities using directly sampled eDNA, to both conventional and eDNA-based xenomonitoring methods where estimates of schistosome abundance require snail density estimates from searches and tests of snail infection status.

It might not be possible to reliably quantify snail infection rates if snails are not readily shedding cercariae [12, 33], and although we succeeded in using the eDNA-based xenomonitoring method with the schistosome hosts *B. pfeifferi* and *B. globosus*, the effectiveness for other host species, perhaps with specific habitat requirements, is unknown. For example, in Lake Malawi the endemic *Bulinus nyassanus*, a host of *S. haematobium*, is found in the upper 2–3 cm of the sediment on open sandy shorelines [41–43]. Additionally, the eDNA testing of *Schistosoma* species may be compromised by hybridization among species, as observed between the closely related *S. mansoni* and *S. rodhaini* [36]. Previous work has demonstrated that hybridization can result in sharing of mitochondrial haplotypes, and distinguishing between the two species at some locations would therefore be intractable using any methods that rely exclusively on amplification of targeted fragments of the mitochondrial genome. A final consideration is the evidence of some cross-species amplification within assays, so in some situations where closely related schistosome species coexist then primers and probes may need to be considered carefully, and potentially with bespoke designs.

In 2012, the World Health Assembly resolved to continue efforts to eliminate schistosomiasis through control and surveillance measures. It is widely recognized that there are multiple required facets to an integrated control programme, focused on both treatment of existing human infections with chemotherapy in synergy with intervention techniques focused on the life stages of the intermediate gastropod host [2, 6]. Physical measures to reduce habitat for snail populations could be used more widely, for example cementing irrigation canals, draining wetlands, or aiming to eliminate snail populations by applying molluscicides (such as new niclosamide formulations) and biological control (including intentional introductions of competitor snails or snail predators). However, in practice such snail control methods may not be practical or ethical in many environmental circumstances. Regardless, the surveillance of freshwaters can enable an early warning of infection risk, and may prove increasingly critical for prevention of reinfection during the elimination phase of control programmes [20, 23], as well as the early detection and elimination of new foci of infection resulting from environmental change [44].

A key consideration for further development of the methods outlined in this study would be to evaluate their effectiveness across different host gastropod species and in different environments. We may expect, for example, that the rate of schistosome cercarial production would vary among sites dependent upon the environmental conditions, for example the temperature and light regimes of assay containers. Therefore, to evaluate potential for effective application, the method needs to be refined enabling robust and systematically consistent results. Moreover, the method would not necessarily be appropriate where snails are rare or hard to sample, and instead in those circumstances eDNA sampling of water collected directly from the natural environment may be more appropriate. Additionally, consideration should be given to the need to standardise training and materials typically available for sampling and analysis in endemic areas [20, 45].

## Conclusions

Here we provide evidence that eDNA-based assays can determine the presence of schistosomes in intermediate host snail species. We suggest that the methods may be appropriate for epidemiological studies and large-scale monitoring in some endemic areas. They could prove useful, alongside other surveillance methods, to inform schistosomiasis control programmes by highlighting freshwater bodies where there is a risk of schistosomiasis transmission. However, further comparative investigations are needed to evaluate the power and practicality of this method in the field, alongside the range of other diagnostic and surveillance methods available.

## Supplementary information

**Additional file 1: Table S1.** Results of *S. mansoni* and *S. haematobium* qPCR experiments, including Cq scores and eDNA copies for all replicates in the experiment.

## Data Availability

The dataset supporting the conclusions of this article is included within the article and its additional files.

## Abbreviations

DNA: Deoxyribonucleic acid
eDNA: Environmental DNA
PCR: Polymerase chain reaction
qPCR: Quantitative polymerase chain reaction
Cq: Quantification cycle
LOD: Limit of detection
LOQ: Limit of quantification
LAMP: Loop-mediated isothermal amplification
