# Supplementary material for: Environmental DNA-based xenomonitoring for determining Schistosoma presence in tropical freshwaters
Source: Parasit Vectors. 2020 Feb 12;13:63. doi: 10.1186/s13071-020-3941-6 (PMC7017522; doi:10.1186/s13071-020-3941-6)
Supplement: Supplementary file 1 — Additional file 1: Table S1. Results of S. mansoni and S. haematobium qPCR experiments, including Cq scores and eDNA copies for all replicates in the experiment. [file 13071_2020_3941_MOESM1_ESM.docx]

**Additional file 1: Table S1.** Results of *S. mansoni* and *S. haematobium* qPCR experiments, including Ct scores and eDNA copies for all replicates in the experiment. (*) indicates no amplification was observed.

|  | *S. mansoni* | *S. mansoni* | *S. mansoni* | *S. mansoni* | *S. haematobium* | *S. haematobium* | *S. haematobium* | *S. haematobium* |
| --- | --- | --- | --- | --- | --- | --- | --- | --- |
| Treatment | replicate | amplification | Ct | eDNA copies | replicate | amplification | Ct | eDNA copies |
| A | 1 | a | 32.51 | 203.787 | 1 | a | 32.92 | 19.547 |
| A | 1 | b | 32.6 | 191.53 | 1 | b | 32.7 | 22.696 |
| A | 1 | c | 32.3 | 239.133 | 1 | c | 33.02 | 18.168 |
| A | 2 | a | 35.21 | 27.315 | 2 | a | 34.61 | 5.976 |
| A | 2 | b | 36.15 | 13.525 | 2 | b | 34.67 | 5.725 |
| A | 2 | c | 34.03 | 66.024 | 2 | c | 34.31 | 7.361 |
| A | 3 | a | 33.85 | 75.46 | 3 | a | 35.09 | 4.264 |
| A | 3 | b | 34.3 | 53.661 | 3 | b | 37.13 | 1.017 |
| A | 3 | c | 33.62 | 89.078 | 3 | c | * | 0 |
| A | 4 | a | 37.19 | 6.233 | 4 | a | 32.58 | 24.754 |
| A | 4 | b | 36.83 | 8.15 | 4 | b | 32.05 | 35.926 |
| A | 4 | c | 37.3 | 5.743 | 4 | c | 32.44 | 27.221 |
| A | 5 | a | 38.42 | 2.478 | 5 | a | * | 0 |
| A | 5 | b | 39.65 | 0.994 | 5 | b | * | 0 |
| A | 5 | c | 37.71 | 4.223 | 5 | c | * | 0 |
| A | 6 | a | 38.93 | 1.707 | 6 | a | 37.98 | 0.562 |
| A | 6 | b | 38.19 | 2.954 | 6 | b | * | 0 |
| A | 6 | c | 38.31 | 2.697 | 6 | c | 36.57 | 1.506 |
| B | 1 | a | 36.57 | 9.857 | 1 | a | 28.78 | 355.084 |
| B | 1 | b | 36.54 | 12.451 | 1 | b | 29.1 | 283.12 |
| B | 1 | c | 36.54 | 8.301 | 1 | c | 28.84 | 339.692 |
| B | 2 | a | 34.59 | 43.167 | 2 | a | 36.72 | 1.354 |
| B | 2 | b | 33.65 | 87.042 | 2 | b | 35.83 | 2.54 |
| B | 2 | c | 35.17 | 28.116 | 2 | c | * | 0 |
| B | 3 | a | 35.31 | 26.416 | 3 | a | 32.65 | 25.936 |
| B | 3 | b | 35.22 | 28.047 | 3 | b | 33.21 | 17.635 |
| B | 3 | c | 34.57 | 45.337 | 3 | c | 33.44 | 15.008 |
| B | 4 | a | 33.73 | 83.247 | 4 | a | 29.46 | 236.258 |
| B | 4 | b | 33.71 | 84.784 | 4 | b | 29.74 | 195.418 |
| B | 4 | c | 34.1 | 63.815 | 4 | c | 29.46 | 236.5 |
| B | 5 | a | 31.46 | 436.744 | 5 | a | 30.38 | 125.687 |
| B | 5 | b | 31.55 | 409.704 | 5 | b | 29.57 | 220.336 |
| B | 5 | c | 31.39 | 459.35 | 5 | c | 30.27 | 135.174 |
| B | 6 | a | 33.28999502 | 115.161 | 6 | a | 32.82 | 23.055 |
| B | 6 | b | 33.99 | 68.836 | 6 | b | 32.9 | 21.877 |
| B | 6 | c | 33.48 | 100.302 | 6 | c | 33.59 | 13.57 |
| C | 1 | a | 35.88 | 17.406 | 1 | a | 33.87 | 11.209 |
| C | 1 | b | 37.48 | 5.409 | 1 | b | 36.86 | 1.404 |
| C | 1 | c | 35.05999775 | 31.671 | 1 | c | 33.84 | 11.403 |
| C | 2 | a | 35.12 | 30.417 | 2 | a | * | 0 |
| C | 2 | b | 34.16 | 61.161 | 2 | b | * | 0 |
| C | 2 | c | 35.16 | 29.509 | 2 | c | * | 0 |
| C | 3 | a | 39.85 | 0.96 | 3 | a | 37.46 | 0.927 |
| C | 3 | b | 39.47 | 1.26 | 3 | b | 37.99 | 0.64 |
| C | 3 | c | 39.71 | 1.06 | 3 | c | 35.83 | 2.871 |
| C | 4 | a | 33.71 | 85.061 | 4 | a | 35.04 | 4.979 |
| C | 4 | b | 34.11 | 63.124 | 4 | b | 34.85 | 5.629 |
| C | 4 | c | 34.32 | 54.279 | 4 | c | 35.27 | 4.25 |
| C | 5 | a | 35.86 | 23.054 | 5 | a | 24.23 | 8118.348 |
| C | 5 | b | 35.23 | 36.099 | 5 | b | 24.15 | 8543.7 |
| C | 5 | c | 37.55 | 6.932 | 5 | c | 24.13 | 8673.707 |
| C | 6 | a | 35.91 | 22.302 | 6 | a | 28.39 | 429.959 |
| C | 6 | b | 36.09 | 19.579 | 6 | b | 29.34 | 220.853 |
| C | 6 | c | 36.1 | 19.494 | 6 | c | 28.58 | 377.916 |
| D | 1 | a | 34.52 | 59.795 | 1 | a | 33.47 | 12.004 |
| D | 1 | b | 35.06 | 40.606 | 1 | b | 35.24 | 3.457 |
| D | 1 | c | 35.51 | 29.641 | 1 | c | 34.16 | 7.376 |
| D | 2 | a | 37.1 | 9.586 | 2 | a | 29.61 | 182.627 |
| D | 2 | b | 35.26 | 35.215 | 2 | b | 29.97 | 142.015 |
| D | 2 | c | 35.3 | 34.251 | 2 | c | 29.37 | 216.899 |
| D | 3 | a | 36.38 | 15.963 | 3 | a | 31.52 | 47.662 |
| D | 3 | b | 36.12 | 19.167 | 3 | b | 31.85 | 37.72 |
| D | 3 | c | 39.28 | 2.04 | 3 | c | 31.09 | 64.019 |
| D | 4 | a | 35.21 | 36.52 | 4 | a | 27.83 | 642.186 |
| D | 4 | b | 35.65 | 26.703 | 4 | b | 28.11 | 525.217 |
| D | 4 | c | 36.02 | 20.583 | 4 | c | 27.77 | 666.481 |
| D | 5 | a | 37.92 | 5.356 | 5 | a | 35.92 | 2.146 |
| D | 5 | b | 38.13 | 4.614 | 5 | b | 35.65 | 2.583 |
| D | 5 | c | 37.81 | 5.767 | 5 | c | 34.51 | 5.764 |
| D | 6 | a | 35.85 | 23.235 | 6 | a | 31.41 | 51.413 |
| D | 6 | b | 36.77 | 12.074 | 6 | b | 31.29 | 55.946 |
| D | 6 | c | 35.5 | 29.652 | 6 | c | 31.39 | 51.896 |
| E | 1 | a | * | 0 | 1 | a | * | 0 |
| E | 1 | b | * | 0 | 1 | b | * | 0 |
| E | 1 | c | * | 0 | 1 | c | * | 0 |
| E | 2 | a | * | 0 | 2 | a | * | 0 |
| E | 2 | b | * | 0 | 2 | b | * | 0 |
| E | 2 | c | * | 0 | 2 | c | * | 0 |
| E | 3 | a | * | 0 | 3 | a | * | 0 |
| E | 3 | b | * | 0 | 3 | b | * | 0 |
| E | 3 | c | * | 0 | 3 | c | * | 0 |
| E | 4 | a | * | 0 | 4 | a | * | 0 |
| E | 4 | b | * | 0 | 4 | b | * | 0 |
| E | 4 | c | * | 0 | 4 | c | * | 0 |
| E | 5 | a | * | 0 | 5 | a | * | 0 |
| E | 5 | b | * | 0 | 5 | b | * | 0 |
| E | 5 | c | * | 0 | 5 | c | * | 0 |
| E | 6 | a | * | 0 | 6 | a | * | 0 |
| E | 6 | b | * | 0 | 6 | b | * | 0 |
| E | 6 | c | * | 0 | 6 | c | * | 0 |
| F | 1 | a | * | 0 | 1 | a | * | 0 |
| F | 1 | b | * | 0 | 1 | b | * | 0 |
| F | 1 | c | * | 0 | 1 | c | * | 0 |
| F | 2 | a | * | 0 | 2 | a | * | 0 |
| F | 2 | b | * | 0 | 2 | b | * | 0 |
| F | 2 | c | * | 0 | 2 | c | * | 0 |
